# Do bark beetle outbreaks amplify or dampen future bark beetle disturbances in Central Europe?

**DOI:** 10.1111/1365-2745.13502

**Published:** 2020-10-12

**Authors:** Andreas Sommerfeld, Werner Rammer, Marco Heurich, Torben Hilmers, Jörg Müller, Rupert Seidl

**Affiliations:** ^1^ Institute of Silviculture University of Natural Resources and Life Sciences (BOKU) Vienna Austria; ^2^ Ecosystem Dynamics and Forest Management Group School of Life Sciences Technical University of Munich Freising Germany; ^3^ Bavarian Forest National Park Grafenau Germany; ^4^ Chair of Wildlife Ecology and Wildlife Management University of Freiburg Freiburg Germany; ^5^ Chair of Forest Growth and Yield Science School of Life Sciences Weihenstephan Technical University of Munich Freising Germany; ^6^ Department of Animal Ecology and Tropical Biology University of Würzburg Würzburg Germany; ^7^ Berchtesgaden National Park Berchtesgaden Germany

**Keywords:** climate change, disturbance interactions, diversity, forest composition, forest structure, iLand

## Abstract

1. Bark beetle outbreaks have intensified in many forests around the globe in recent years. Yet, the legacy of these disturbances for future forest development remains unclear. Bark beetle disturbances are expected to increase further because of climate change. Consequently, feedbacks within the disturbance regime are of growing interest, for example, whether bark beetle outbreaks are amplifying future bark beetle activity (through the initiation of an even‐aged cohort of trees) or dampening it (through increased structural and compositional diversity).
2. We studied bark beetle–vegetation–climate interactions in the Bavarian Forest National Park (Germany), an area characterised by unprecedented bark beetle activity in the recent past. We simulated the effect of future bark beetle outbreaks on forest structure and composition and analysed how disturbance‐mediated forest dynamics influence future bark beetle activity under different scenarios of climate change. We used process‐based simulation modelling in combination with machine learning to disentangle the long‐term interactions between vegetation, climate and bark beetles at the landscape scale.
3. Disturbances by the European spruce bark beetle were strongly amplified by climate change, increasing between 59% and 221% compared to reference climate. Bark beetle outbreaks reduced the dominance of Norway spruce (*Picea abies* (L.) Karst.) on the landscape, increasing compositional diversity. Disturbances decreased structural diversity within stands (*α* diversity) and increased structural diversity between stands (*β* diversity). Overall, disturbance‐mediated changes in forest structure and composition dampened future disturbance activity (a reduction of up to −67%), but were not able to fully compensate for the amplifying effect of climate change.
4. *Synthesis*. Our findings indicate that the recent disturbance episode at the Bavarian Forest National Park was caused by a convergence of highly susceptible forest structures with climatic conditions favourable for bark beetle outbreaks. While future climate is increasingly conducive to massive outbreaks, the emerging landscape structure is less and less likely to support them. This study improves our understanding of the long‐term legacies of ongoing bark beetle disturbances in Central Europe. It indicates that increased diversity provides an important dampening feedback, and suggests that preventing disturbances or homogenizing post‐disturbance forests could elevate the future susceptibility to large‐scale bark beetle outbreaks.

## Graphical abstract

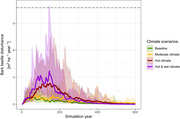

## 1 INTRODUCTION

Disturbances are key drivers of the structure, composition and functioning of forest ecosystems (Turner, 2010). Disturbance regimes are strongly driven by climatic conditions, and are thus sensitive to ongoing global climate change (Seidl et al., 2017). Due to increasing disturbances, the interactions and feedbacks within disturbance regimes are also of increasing importance (Buma, 2015). Insect outbreaks are biotic disturbances of major importance in forests around the globe (Anderegg et al., 2015). In the temperate and boreal biome, bark beetles are the most important insect disturbance agents (Netherer & Schopf, 2010; Raffa, Grégoire, & Lindgren, 2015). Bark beetle outbreaks are particularly affected by climate change due to the ectothermic physiology of the beetles (Jakoby, Lischke, & Wermelinger, 2019) and the drought sensitivity of the defence system of trees (Huang et al., 2020). Consequently, bark beetle outbreaks are intensifying in many forests globally (Hicke, Meddens, & Kolden, 2016; Marini et al., 2017).

The European spruce bark beetle (*Ips typographus* L., hereafter referred to as ‘bark beetle’ for brevity) is the economically most important bark beetle species in conifer forests of Europe, primarily attacking Norway spruce (*Picea abies* (L.) Karst., hereafter referred to as ‘spruce’) trees. At low population levels, bark beetles preferentially colonize weakened trees. Favourable conditions, like drought that weakens tree defences, high temperatures that accelerate bark beetle population growth and major windthrows that provide large amounts of breeding material, can trigger large‐scale outbreaks, resulting in widespread mortality of healthy spruce trees (Biedermann et al., 2019; Kausrud et al., 2011; Marini et al., 2017). Spruce is a tree species of major economic interest in Europe (Grégoire, Raffa, & Lindgren, 2015), and growth in monocultures was promoted by foresters in the past (Hlásny et al., 2019), which further increases the susceptibility of the current vegetation to bark beetle outbreaks. On average, 14.5 Mill. m^3^ of timber were affected by bark beetle disturbance annually between 2002 and 2010 in Europe (Seidl, Schelhaas, Rammer, & Verkerk, 2014), and bark beetles were an important driver of the recent doubling in canopy mortality across Central Europe (Senf et al., 2018).

Post‐disturbance forest development of areas affected by bark beetles is receiving increasing attention as bark beetle outbreaks in forest landscapes of Central Europe increase in frequency and severity. Most of the recently disturbed forests regenerate vigorously even in the absence of human intervention, with several thousand saplings colonizing post‐outbreak areas one to two decades after disturbance (Senf, Müller, & Seidl, 2019; Wild et al., 2014; Zeppenfeld et al., 2015). Interestingly, recent studies show that spruce, which is a mid‐ to late‐seral species adopted to cool climate, dominates the regenerating cohort in subalpine forests, while pioneer species and warm‐adapted species are largely missing (Macek et al., 2017). This suggests that recent disturbances have not catalysed tree species change (cf. Thom, Rammer, & Seidl, 2017a, 2017b), and that forests will likely recover to a species composition that is similar to the pre‐disturbance state. Given the expectation of continued warming in coming decades (Stocker et al., 2013) and the large, even‐aged, spruce‐dominated cohort regenerating on the landscape, it has been hypothesized that the recent wave of disturbances will increase forest susceptibility to future disturbances. This is supported by the observation that past waves of disturbance also contribute to current disturbance activity in Europe's forests (Schurman et al., 2018). An alternative hypothesis is that forests regenerating following natural disturbances are *born complex* (Donato, Campbell, & Franklin, 2012), that is, they have high structural diversity despite being of similar age. Complexity can arise from the many biological legacies left after natural disturbances (e.g. remnant live trees, standing and downed woody debris, advanced tree regeneration not affected by disturbance, heterogeneous seed bank) and their influence on post‐disturbance forest development (Diskin, Rocca, Nelson, Aoki, & Romme, 2011; Johnstone et al., 2016; Kayes & Tinker, 2012; Seidl, Rammer, & Spies, 2014). This complexity can result in multiple successional pathways (Meigs et al., 2017; Tepley, Swanson, & Spies, 2013) creating diversity on the landscape, which, in turn, dampens future forest susceptibility to spreading disturbances, such as bark beetles (Hart, Veblen, Mietkiewicz, & Kulakowski, 2015; Honkaniemi, Rammer, & Seidl, 2020; Seidl, Donato, Raffa, & Turner, 2016). Empirical findings and simulation studies from North America suggest that past bark beetle outbreaks exert strong negative feedbacks on subsequent outbreaks (Hart et al., 2015; Kashian, Jackson, & Lyons, 2011; Temperli, Veblen, Hart, Kulakowski, & Tepley, 2015). These negative feedbacks emerge mainly due to shifts in tree species composition and subsequent reduction in host availability as well as increased structural diversity (Kayes & Tinker, 2012; Temperli et al., 2015). Recent analyses for Central Europe show that natural forests are characterized by higher structural diversity than managed forests in the first decades after disturbance (Senf et al., 2019). Yet, it remains unclear whether such a disturbance‐mediated increase in diversity will persist as forests mature and develop through a stem exclusion stage. Furthermore, whether elevated diversity is able to counteract the increasing disturbance pressure caused by climate change remains unclear (Dobor et al., 2020; Temperli, Bugmann, & Elkin, 2013; Thom et al., 2017b).

Testing hypotheses on the long‐term development of forest landscapes under future climate change relies on the use of simulation models. Dynamic process‐based simulation models represent key biological processes of forest dynamics (e.g. competition, growth, regeneration, mortality, seed dispersal, disturbance) based on first principles of ecology (Gustafson, 2013), and are thus able to make robust projections of future vegetation development. Process‐based simulation models can investigate the complex interdependencies of interacting disturbance agents, disturbance legacies and the compositional and structural diversity of forest landscapes under past and future climatic conditions. Analysing the feedbacks of disturbances on vegetation development and their implications for future disturbances particularly relies on the use of simulation models, due to the need to consider time horizons of multiple centuries (Temperli et al., 2013; Thom, Rammer, Garstenauer, & Seidl, 2018). A further advantage of using simulation modelling is the ability to derive dynamic reference trajectories (e.g. simulations of undisturbed forest development) in order to isolate the effects of focal processes such as disturbances (Dobor et al., 2018). Additionally, while the investigation of disturbances necessitates analyses across landscapes, field experimentation with sufficient replication is not possible at this spatial scale (Phillips, 2007), a problem that can be overcome by conducting experiments in silico.

Here we used landscape‐scale simulation modelling to investigate how bark beetle outbreaks influence structural and compositional diversity, and how they affect future bark beetle dynamics under climate change. We hypothesized that bark beetle disturbances will increase under future climate conditions (H1). Specifically, we expected that higher temperatures will accelerate the development of bark beetles and that associated increases in water demand will increase the susceptibility of host trees (Huang et al., 2020; Netherer & Schopf, 2010; Seidl & Rammer, 2017). Furthermore, we hypothesized that future bark beetle disturbances will increase compositional and structural diversity of forest landscapes (H2). We expected compositional diversity to increase due to disturbances creating niches for the establishment of new tree species, and due to reduction in the competitive strength of spruce (Hilmers et al., 2019; Thom et al., 2017a). Furthermore, we expected forest structure to increase in diversity due to the effect of disturbance legacies as well as spatially heterogeneous regeneration and stand development (Donato et al., 2012; Meigs et al., 2017). Finally, we quantified the feedbacks of disturbance‐mediated compositional and structural diversity on future bark beetle outbreaks, hypothesizing a dampening (self‐regulating) effect of forest dynamics under disturbance (H3). We expected that more diverse forests will dampen future bark beetle disturbances due to the presence of fewer host trees and decreased host connectivity (Seidl, Müller, et al., 2016; Temperli et al., 2013).

## 2 MATERIALS AND METHODS

### 2.1 Study landscape

**FIGURE 1:**
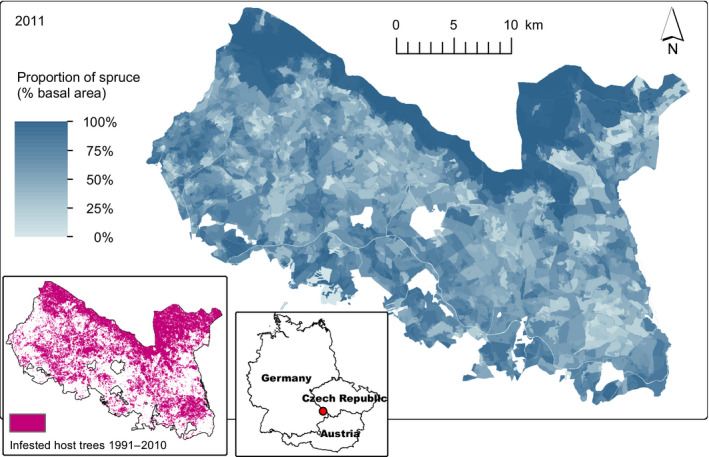
The Bavarian Forest National Park landscape, depicting the proportion of Norway spruce (i.e. the host tree of the European spruce bark beetle) on total basal area in the year 2011 (i.e. the initial year of this study). The lower left panel shows bark beetle infested host trees between 1991 and 2010 and the lower right panel indicates the location of the landscape in Central Europe

The Bavarian Forest National Park (BFNP) is a forested landscape in Southeastern Germany (Figure 1). The area is characterized by moderate topography, with an elevation range from 655 to 1,420 m a.s.l. Mean annual temperature ranges from 3.5 to 7.0°C and decreases with elevation, and annual precipitation ranges from 1,000 to 1,900 mm, increasing with elevation. Dominant tree species include Norway spruce at mid‐ to high elevations as well as silver fir (*Abies alba* Mill.) and European beech (*Fagus sylvatica* L.) mixed with spruce at lower elevations. Natural tree species composition was heavily affected by humans at least since 500 BC, which has reduced the proportion of silver fir (van der Knaap et al., 2020). The cultivation of spruce was expanded in the 16th century due to an increasing wood demand for manufacturing, and in the 19th century due to the emergence of commercial forestry. Founded in 1970, the BFNP is Germany's oldest national park. The 24,000 ha landscape was protected in two phases, with an extension of the initial protected area in 1997. We here focus on the 13,985 ha designated as national park in 1970 due to data availability issues. The BFNP saw the largest unsuppressed bark beetle outbreak in Central Europe in recent history, characterized by two distinct outbreak waves (1996–2000 and 2005–2009) affecting close to 50% of the study area.

### 2.2 Simulation model

We used the individual‐based forest landscape and disturbance model iLand (Seidl, Rammer, Scheller, & Spies, 2012) to study the effects of future climate and disturbance regimes. Specifically, we applied the model to quantify the interactions between structural and compositional diversity at BFNP and the development of bark beetle populations. iLand dynamically simulates the regeneration, growth and mortality of individual trees, influenced by climate, soil, initial legacies of vegetation and disturbance. The spatial grain of the simulations is 2 m × 2 m for the calculation of the light regime, while ecosystem processes (e.g. water and carbon cycles) are tracked at a grain of 100 m × 100 m; the spatial extent of the simulations is 13,985 ha of BFNP. We simulated disturbances by bark beetle outbreaks and windthrow, the two most important disturbance agents in the region, explicitly. Simulated bark beetle population dynamics accounts for beetle development and phenology and models beetle dispersal in a spatially explicit manner. The processes of host colonization, tree defence and temperature‐dependent winter mortality are also considered (Seidl & Rammer, 2017). As the ability to realistically simulate bark beetle outbreaks is crucial for the current study, we extensively tested spatial and temporal patterns of simulated bark beetle infestations against independent data (Kautz, Dworschak, Gruppe, & Schopf, 2011; Figures S1–S3). In addition, we examined model performance in a pattern‐oriented modelling approach (Grimm et al., 2005), comparing simulated data against independent empirical observations of tree growth and potential natural vegetation (PNV) development (Figures S4–S8). A more detailed description of iLand can be found in Seidl, Spies, et al. (2012) and Thom et al. (2017b). The model code and executable as well as an extensive online documentation are available at http://iLand.boku.ac.at.

### 2.3 Initial conditions and climate scenarios

Simulation runs were initiated with the state of the vegetation in 2011, that is, after the most recent bark beetle outbreak waves of the years 1996–2010. We initialized the model based on the latest available plot‐level forest inventory data regarding tree species, basal area, tree height and stand age (year 1992), and prescribed bark beetle disturbances as observed from aerial surveys (Kautz et al., 2011) to obtain the initial vegetation state (year 2011) from dynamic simulations. Data on soils and climate were available at a 100 m × 100 m grid. Soil properties required in the simulation (i.e. effective soil depth, the relative proportion of sand, silt and clay, as well as plant‐available nitrogen and the initial carbon stocks in litter and soil organic matter layers) were derived by combining the wall‐to‐wall site classification system of BFNP with quantitative soil profile data.

We ran simulations under four different climate scenarios. Historical climate data from 1980 to 2015 was used as a reference period for constructing a baseline climate scenario by randomly drawing years with replacement. In addition, we simulated three different climate change scenarios, representing different combinations of representative concentration pathways (RCP 4.5 and 8.5) and climate models (ICHEC‐EC‐EARTH and MOHC‐HadGEM2‐ES). The temperature and precipitation changes resulting from these scenarios in our study region are detailed in Table S1. In the following text, we refer to the climate scenarios as baseline climate scenario (BC), moderate climate change scenario (MC, RCP 4.5 ICHEC‐EC‐EARTH), hot climate change scenario (HC, RCP 8.5, ICHEC‐EC‐EARTH) and hot and wet climate change scenario (HWC, RCP 8.5, MOHC‐HadGEM2‐ES). Climate change time series were extended beyond 2099 by sampling with replacement from the years 2070–2099. The dependency of our simulation results on this particular approach of generating a long‐term climate time series was tested in a sensitivity analysis (see Figure S8). All climate data were statistically downscaled to the study region at 100 m horizontal resolution (see Seidl et al., 2019 for details). As the current generation of climate models is not yet able to capture extreme local wind events well, the occurrence of storm events was based on historically observed wind data, assuming no changes in future peak wind speeds and return intervals. To isolate the effect of disturbances on forest structure and composition, we simulated two different disturbance scenarios in each climate scenario, that is, an undisturbed control scenario for which disturbances were omitted throughout the entire simulation period (referred hereafter as *undisturbed*) and a scenario in which disturbances and their impacts on vegetation were dynamically simulated in iLand (referred hereafter as *disturbed*). All simulations were replicated 20 times to account for stochasticity.

### 2.4 Analyses

In order to assess the long‐term consequences of disturbances we simulated forest development over a period of 600 years. First, to test our hypothesis of increased future bark beetle disturbances (H1), we evaluated the amount of growing stock disturbed by beetles in the different simulation scenarios. To put simulated future trajectories into context, values were compared to reference data for the outbreaks of the period 1996–2010.

Second, to address our hypothesis on disturbance‐mediated increases in diversity (H2), we compared *undisturbed* simulations to those simulating wind and bark beetle disturbances dynamically (*disturbed*). We here characterized diversity at two spatial scales (stand [100 m grid cells], landscape) and for two domains (structure, composition), deriving a total of eight indicators of diversity (see Table 1) from the simulated data at 50‐year time steps. For landscape‐level analyses, an eight‐neighbour rule was used to define neighbourhood. To attribute individual effects of climate change, disturbances and the combined effect of climate change and disturbances, we compared diversity indicators between contrasting trajectories. Our reference simulations without climate change and disturbances (i.e. BC and *undisturbed*) were contrasted to simulations with climate change only (e.g. HWC and *undisturbed*), with disturbances only (i.e. BC and *disturbed*) and to simulations with combined effects of climate change and disturbances (e.g. HWC and *disturbed*). We used principle component analyses (PCA), as implemented in the R package *factoextra* (version 1.0.5; Kassambara & Mundt, 2017), to reduce the dimensions of our dataset and visualize differences in forest development trajectories between *disturbed* and *undisturbed* simulations.

**TABLE 1:** Overview of the indicators analysed

Attribute	Scale	Indicator (abbreviation)	Unit	Description
Structure	Stand	*α*‐diversity of tree height (AlpHei)	Dim.	Shannon–Wiener index of height classes (4 m class width) within forest stands
	Stand	*α*‐diversity of tree diameter (AlpDbh)	Dim.	Shannon–Wiener index of dbh classes (4 cm class width) within forest stands
	Landscape	Canopy cover (CanCov)	Dim.	Horizontal structure and distribution of canopy, described by the proportion of the ground surface that is covered by tree crowns
	Landscape	Rumple index (RumInd)	Dim.	Vertical structure and distribution of canopy, described by the ratio of the canopy surface area to the ground surface area
	Landscape	*β*‐diversity of tree height (BetHei)	Dim.	Shannon–Wiener index of height classes (4 m class width) among forest stands, multiplicative beta diversity (*β*‐div. = *γ*‐div./*α*‐div.)
	Landscape	*β*‐diversity of tree diameter (BetDbh)	Dim.	Shannon–Wiener index of dbh classes (4 cm classes) among forest stands, multiplicative beta diversity (*β*‐div. = *γ*‐div./*α*‐div.)
Composition	Landscape	Proportion of spruce (ProSpr)	%	Per cent of Norway spruce on the total basal area of all trees
	Landscape	Aggregation index of potential host trees for bark beetle attack (AggInd)	%	Percentage of the landscape with contiguous raster cells hosting spruce trees above 15 cm dbh

Abbreviations: Dbh, diameter at breast height; Dim., dimensionless.

Third, we used simulation outputs in combination with machine learning to quantify the effect of differences in diversity on simulated bark beetle outbreaks (H3). Specifically, we trained a random forest model (Breiman, 2001) using the r package randomForest (version 4.6‐14; Liaw & Wiener, 2002), on the simulation outcomes from the *disturbed* simulation series, using the amount of growing stock disturbed by bark beetles as the response variable and the indicators of structural and compositional diversity (cf. Table 1) as well as climate data (e.g. mean annual temperature, mean annual precipitation) as predictors. To quantify the effect of disturbance‐mediated changes in forest structure and composition on bark beetle dynamics, we predicted the amount of growing stock affected by bark beetles using the random forest model. Specifically, predictions were made for (a) the forest structure and composition simulated in the *disturbed* series and (b) the forest structure and composition simulated in the *undisturbed* series, using the same random forest model. This approach allowed us to disentangle the effects of disturbance‐mediated changes in forest structure and composition on the propensity of bark beetle disturbances, effectively quantifying potential self‐regulating feedbacks within the disturbance regime. We used the R software for statistical computing (version 3.5.1; R Core Development Team, 2018) for data preparation, analyses of simulation data and visualization.

## 3 RESULTS

### 3.1 Future bark beetle dynamics

**FIGURE 2:**
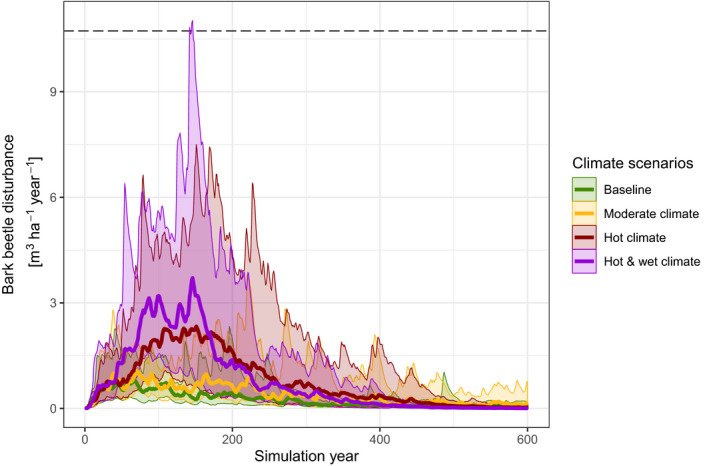
Projected growing stock affected by bark beetles in different climate scenarios. The figure shows the exponential moving average of bark beetle disturbance (averaging window = 10 years with reduction factor of 1/10). Bold lines are mean values and envelopes indicate the range of values derived from 20 replicated simulations per scenario. The horizontal dashed line at *y* = 10.73 indicates the 10‐year exponential moving average of bark beetle activity in the recent past (1990–2010)

Climate change had a strong amplifying effect on future bark beetle dynamics. The cumulative growing stock affected by bark beetles was 59.0% higher under moderate climate change scenario, and 204.8% and 221.1% higher in the hot and hot and wet climate change scenarios, respectively, compared to baseline climate. These cumulative differences between climate scenarios mainly accrued in the first half of the 600‐year simulation period, while differences among climate scenarios diminished in the last century of the simulation. The general pattern of future bark beetle dynamics over time was similar in all climate scenarios. Bark beetle activity was low in the first years of the analysis, and peaked between years 50 and 200 of the simulation. Of the cumulative growing stock affected by bark beetle over the 600‐year study period, between 57.6% (scenario MC) and 85.6% (scenario HWC) accrued in the first 200 years of the simulation (Figure 2). Outbreak patterns within this period differed strongly with climate scenario. Under the HWC scenario, bark beetle outbreaks peaked early and showed the highest peaks (10‐year moving average of up to 3.71 m^3^ of growing stock disturbed per ha and year).The hot climate scenario resulted in a lower and more prolonged peak (maximum 10‐year moving average of 2.30 m^3^ ha^−1^ year^−1^). While the central tendency in both of these scenarios was thus below the bark beetle impact observed for the outbreaks of 1990–2010 (10.73 m^3^ ha^−1^ year^−1^), beetle activity in one of the 20 replicate simulations under the hot and wet climate scenario exceeded recent observations (Figure 2). Under moderate climate change, future outbreaks led to a distinctly different pattern over time, with more frequent yet much smaller outbreak waves (maximum 10‐year moving average of 1.07 m^3^ ha^−1^ year^−1^) occurring in the simulations.

### 3.2 Effects of bark beetle disturbances on diversity

**FIGURE 3:**
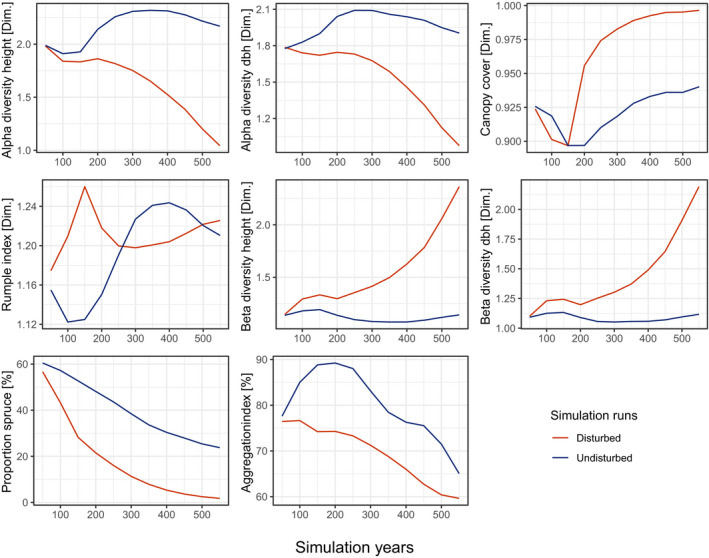
Effect of disturbances on forest structure and composition. Shown are the mean effects for the hot and wet climate scenario, which is the scenario with the highest disturbance activity in terms of timber volume disturbed (cf. Figure 2). For results of all other climate scenarios see Figures S10–S12. We refer to Table 1 for a detailed description of the indicators used

Bark beetle disturbances increased compositional diversity within the landscape (Figure 3). Specifically, they reduced spruce dominance and increased the spatial heterogeneity of species composition. Spruce proportion decreased dramatically under all climate scenarios, even under baseline climate it declined by more than 50% (from an initial value of 60.5% to 28.9% at the end of the simulation period, Figure S10). Spruce virtually disappeared under the hot climate scenarios (only 1.7% of spruce basal area remained under HWC at the end of the simulation period). The most important factor contributing to spruce decline was the amplifying interactions between climate change and bark beetle disturbances. European beech and silver fir were the main tree species replacing spruce on the landscape (Figure S9).

**FIGURE 4:**
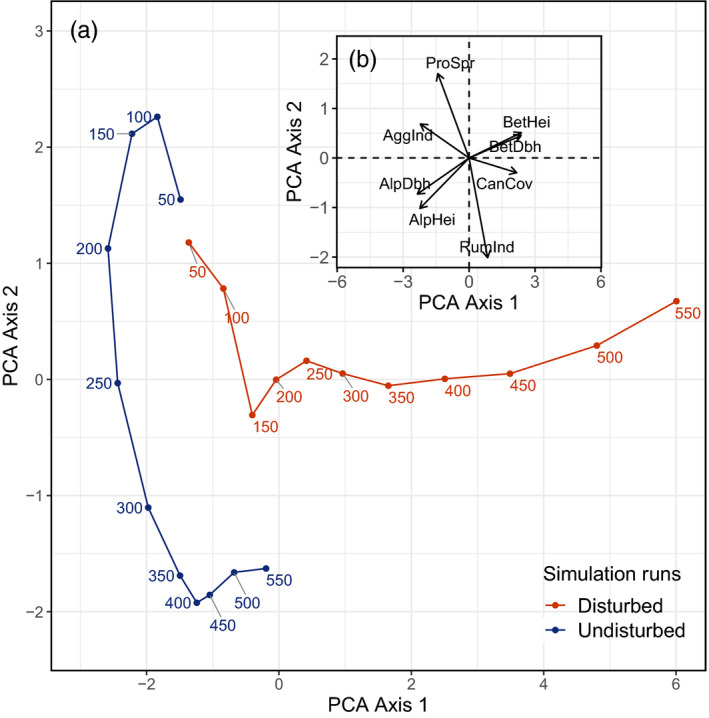
Principle component analysis showing forest development trajectories in *disturbed* and *undisturbed* simulation runs (Panel a). The disturbed trajectory shows the hot and wet climate scenario, which is the scenario with the highest disturbance activity in terms of timber volume disturbed (cf. Figure 2), with numbers indicating simulation years. Panel (b) shows the loadings of individual variables contributing to the PCA (for abbreviations we refer to Table 1)

The response of structural diversity to bark beetle disturbances was less clear and differed with scale. Beta diversity increased and alpha diversity decreased in *disturbed* compared to *undisturbed* simulations. This pattern was consistent for both diameter and height diversity. Among‐stand diversity doubled in simulations under the climate scenario with the most intense bark beetle impacts (HWC), while within‐stand diversity halved over the simulation period compared to *undisturbed* simulations. Similar to the decline of spruce, these changes were primarily driven by the combined effects of climate change and disturbances (Table 2). Principle component analysis suggests that *disturbed* scenarios followed a distinctly different trajectory of forest development compared to *undisturbed* simulations (Figure 4a). The first two principle components explained 90.2% of the total variation in our data (PCA axis 1 = 63.5% and PCA axis 2 = 26.7%). Factor loadings (Figure 4b) showed that variables of forest composition and structure were largely orthogonal, while indicators of *α* and *β* diversity were inversely related. In early years of the simulation, the development trajectories mainly differed in composition (i.e. along the PCA axis 2 in the Figure 4a,b), while in later years divergent structural development (i.e. differences along PCA axis 1) add to increasingly diverging trajectories.

**TABLE 2:** Response of forest structure and composition to climate change and disturbances. Results are shown as differences in indicator values averaged over the entire simulation period, relative to the mean value of simulations with baseline climate and no disturbances. Shown are results for the hot and wet climate scenario, which is the scenario with the highest disturbance activity in terms of timber volume disturbed (cf. Figure 2). For results of all other climate scenarios see Tables S2–S4. See Table 1 for a detailed description of the indicators used

Indicator	Attribute	Scale	Effect of climate change (*undisturbed simulations*)	Effect of disturbances (*disturbed simulations under BC climate*)	Effect of disturbances and climate change
*α*‐diversity height	Structure	Stand	−0.8%	−2.0%	−25.5%
*α*‐diversity dbh		Stand	+2.4%	−2.8%	−20.5%
Canopy cover		Landscape	+2.4%	−0.2%	+7.1%
Rumple index		Landscape	+0.8%	+1.2%	+2.4%
*β*‐diversity height		Landscape	+2.0%	+1.3%	+42.1%
*β*‐diversity dbh		Landscape	+1.3%	+1.4%	+35.2%
Proportion of spruce	Composition	Landscape	−8.2%	−9.4%	−58.9%
Aggregation index		Landscape	+1.7%	−5.3%	−11.6%

### 3.3 Disturbance‐mediated forest development affects future disturbances

**FIGURE 5:**
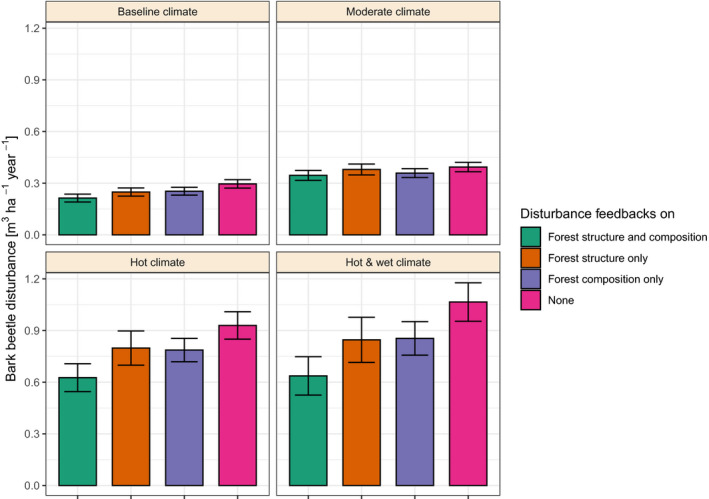
Effects of disturbance‐mediated feedbacks of forest structure and composition on bark beetle activity, summarized over the entire 600‐year simulation period. Disturbance feedbacks were isolated using a random forest model trained on simulation data. Bars indicate mean values and whiskers give the 95% confidence interval over all replicates per climate scenario

We found strong evidence for dampening feedbacks within the disturbance regime, with disturbance‐mediated changes in forest structure and composition decreasing future disturbance activity. The random forest model used to disentangle the effects of forest structure, composition and climate on disturbance activity was well able to describe the simulated data, explaining 96.0% of its variance. Sensitivity analyses using this random forest model showed that due to the disturbance effects on forest structure and composition, the amount of growing stock affected by bark beetles was 67.4% lower than under *undisturbed* forest structure and composition (scenario HWC). Structural effects—although more ambiguous across the indicators investigated (see Figure 3)—had a similar dampening impact as compositional effects (Figure 5). The strength of dampening feedbacks in the disturbance regime increased with disturbance activity and was considerably higher in the hot as well as in the hot and wet climate scenarios compared to moderate and baseline climate. However, dampening feedbacks from disturbance‐mediated changes in forest structure and composition could not fully compensate the disturbance increases caused by climate change.

## 4 DISCUSSION

Natural disturbances from both abiotic and biotic agents have increased strongly in recent decades in many parts of the world (Kautz, Meddens, Hall, & Arneth, 2017; Senf et al., 2018; Westerling, 2016), with unprecedented disturbance episodes affecting landscapes throughout the globe. Given that climate change is an important driver of shifting disturbance regimes (Dale et al., 2001; Marini et al., 2017; Seidl et al., 2017), it is important to quantify potential future trajectories of natural disturbances considering the range of possible future climate conditions. Here we show that climate change has an amplifying effect on future bark beetle outbreaks in Central Europe, supporting our initial hypothesis (H1). This finding is in line with previous analyses for different forest ecosystems in the northern hemisphere (Bentz et al., 2010; DeRose, Bentz, Long, & Shaw, 2013; Dobor et al., 2020; Seidl, Rammer, Jäger, & Lexer, 2008; Seidl, Schelhaas, Lindner, & Lexer, 2009; Temperli et al., 2013; Thom et al., 2017b), emphasizing that bark beetle disturbances are among the most climate‐sensitive processes in temperate forest ecosystems. What makes our study unique is that it focused on the landscape that experienced the largest unmanaged bark beetle outbreak recorded throughout Europe in the recent history. Despite the high climate sensitivity of bark beetle outbreaks, our results suggest that future outbreaks of bark beetles in the BFNP are unlikely to reach similar intensities as those that have occurred in the recent past. While individual simulation trajectories reached previously observed levels of bark beetle activity, the central tendency remained considerably below these levels even under severe climate change (scenarios HC & HWC). This suggests that future climate is increasingly conducive to such massive outbreaks, but the emerging landscape structure is unlikely to support them. Our results thus indicate that the BFNP bark beetle outbreaks of the recent past resulted from the convergence of favourable climate and highly susceptible forest conditions (Aukema et al., 2006; Duan, Taylor, & Fuester, 2011; Seidl, Schelhaas, & Lexer, 2011). These two factors are unlikely to coincide again in the future at BFNP because compositional and structural diversity is increasing relative to the low values of the past, which were largely the result of past land‐use. More broadly the importance of forest structure and composition for bark beetle outbreaks found here is in line with insights from ecosystems in Northern America, where the dampening effects of structure and composition can even exceed the amplifying effect of climate (DeRose et al., 2013; Hart et al., 2015; Temperli et al., 2015).

We showed that the BFNP landscape is dynamically changing away from its recent historical condition of structurally homogeneous and highly spruce‐dominated forests. European beech and silver fir expanded in our simulations, which is in line with previous projections of future forest development (Cailleret, Heurich, & Bugmann, 2014; Hanewinkel, Cullmann, Schelhaas, Nabuurs, & Zimmermann, 2013; Temperli et al., 2013; Thom et al., 2017b). In this context, it is important to note that we did not explicitly consider disturbance agents that target specific tree species other than Norway spruce. Future work should aim to capture the biotic disturbance regime more comprehensively, as other agents could impede the expansion of fir and beech in the future. For bark beetle outbreaks, we show that climate change has both positive (amplifying) and negative (dampening) impacts, fostering beetle development and increasing host susceptibility (direct effect, short‐term), while at the same time, shifting the competitive balance towards non‐host trees (indirect effect, mid‐ to long‐term; Seidl et al., 2017). A second important trend in future forest development was an increase in structural diversity between stands (*β*‐diversity). This suggests that the heterogeneity in diameters and tree heights at a grain of 100 m will considerably increase in the future BFNP landscape (cf. Schall et al., 2018). In contrast to the change in tree species composition—which was primarily driven by climate change—the increase in beta diversity was mainly the result of increasing disturbances. Simultaneously, structural diversity within 100 m grid cells decreased in our simulations. It is important to note that we used Whitaker's multiplicative definition of beta diversity (Jost, 2007), which means that the values for both levels are not independent of each other. The patterns identified here, however, are ecologically realistic, given that most bark beetles only disperse several tens of meters (resulting in spatially clustered mortality; Kautz et al., 2011), and that the mean size of disturbance patches in Central Europe is roughly 1 ha (Senf, Pflugmacher, Hostert, & Seidl, 2017; Sommerfeld et al., 2018).

Overall, bark beetle outbreaks increased the structural and compositional diversity of forest ecosystems in our simulation, supporting our initial hypothesis (H2). These findings are in line with previous studies relating forest composition and structure to disturbances. Panayotov, Kulakowski, Laranjeiro Dos Santos, and Bebi (2011), for instance, using empirical data and remote sensing, related large variation in tree heights and diameters to prior wind disturbances. Janda et al. (2017) documented that past disturbance severity is a strong driver of current stand structure, using a combination of dendroecology and historical data sources. Kayes and Tinker (2012) and Veblen, Hadley, Reid, and Rebertus (1991) found that bark beetles increase the structural and compositional diversity by releasing advanced regeneration and small‐diameter trees. Likewise, Kashian et al. (2011) report increases in stand structural diversity as a result of prior bark beetle outbreaks. Silva Pedro, Rammer, and Seidl (2016) found that disturbances have positive effects on forest composition at both alpha and beta levels even under high disturbance frequencies. The fact that they did not study highly spatially aggregated disturbance agents such as bark beetles might account for the different response of alpha diversity compared to our study. Overall, we show that bark beetle disturbances can substantially alter forest development trajectories. It is thus essential to consider them explicitly in models that aim to project the future of forest ecosystems (Huang et al., 2020).

We documented important dampening feedbacks within the natural disturbance regime of Central Europe's forests. Specifically, we showed that bark beetle disturbances have a negative influence on future bark beetle activity via modifying the structure and composition of forest ecosystems. Dampening feedbacks are well‐documented for many fire‐driven systems, where burning consumes fuel, limiting subsequent fire activity (Bigler, Kulakowski, & Veblen, 2005; Harvey, Donato, & Turner, 2016; Parks, Holsinger, Miller, & Nelson, 2015). By analogy, studies for biotic disturbances have shown dampening feedbacks due to depletion of suitable hosts on the landscape (Cruickshank, Jaquish, & Nemec, 2010; Temperli et al., 2013; Thom et al., 2017b). Engelmann spruce *Picea engelmanni* *i* – spruce beetle *Dendroctonus rufipennis* systems in Northern America, for instance, have reduced susceptibility to large‐scale outbreaks after disturbance due to the paucity of large‐diameter host trees (Hart et al., 2015; Temperli et al., 2015). We highlight how structural diversity can serve as another critical dampening feedback mechanism within biotic disturbance regimes. Our simulations suggest that disturbance‐mediated beta diversity inhibits the spread of future bark beetle outbreaks (see also Honkaniemi et al., 2020). This is in line with recent findings of dampening feedbacks between wildfires and bark beetle outbreaks (Seidl, Donato, et al., 2016). Based on our simulations, the effect size of increased structural diversity was comparable to that of depleted host trees (cf. Figure 5), suggesting that a diverse vertical and horizontal structure between stands is an important factor mitigating bark beetle outbreaks.

Our results indicate that disturbance‐mediated forest development trajectories are less prone to very large‐scale bark beetle outbreaks compared to those in which natural disturbances are absent for a long time. This finding suggests that preventing bark beetle disturbances via technical measures (e.g. trapping, chemicals, timely remove of infested trees) could in fact increase the risk for future, large‐scale bark beetle outbreaks. This result is in line with findings regarding the effect of fire prevention (Stephens et al., 2013), and with recent analyses showing that a management‐induced reduction in bark beetle disturbances can lead to increased disturbances from wind (Dobor et al., 2020). It furthermore supports broader conceptual arguments that suggest preventing natural disturbances is only successful under a limited set of conditions and can lead to unintended consequences (Holling & Meffe, 1996; Seidl, 2014).

We conclude that while future climate change will intensify forest disturbance regimes, potent dampening feedbacks such as disturbance‐mediated increases in diversity exist in the forest ecosystems of Central Europe. Management should aim to support and—where possible—mimic these processes to foster the adaptation of forest ecosystems to changing forest disturbance regimes.

## AUTHORS' CONTRIBUTIONS

A.S., W.R., and R.S. designed the study, analysed the data and wrote the paper; M.H., T.H. and J.M. contributed data and commented on the manuscript. All authors contributed critically to earlier drafts and gave final approval for publication.

### PEER REVIEW

The peer review history for this article is available at https://publons.com/publon/10.1111/1365‐2745.13502.

## Supporting information

Supplementary Material

## Data Availability

Data are available from the figshare repository: https://doi.org/10.6084/m9.figshare.12885200.v1 (Sommerfeld et al., 2020).
