# Supplementary material for: Do bark beetle outbreaks amplify or dampen future bark beetle disturbances in Central Europe?
Source: J Ecol. 2020 Oct 12;109(2):737–49. doi: 10.1111/1365-2745.13502 (PMC7894307; doi:10.1111/1365-2745.13502)
Supplement: Supplementary file 1 — Supplementary Material [file JEC-109-737-s001.docx]

# Supplement: Do bark beetle outbreaks amplify or dampen future bark beetle disturbances in Central Europe?

**Andreas Sommerfeld, Werner Rammer, Marco Heurich, Torben Hilmers, Jörg Müller, Rupert Seidl**

1. Bark beetle tests


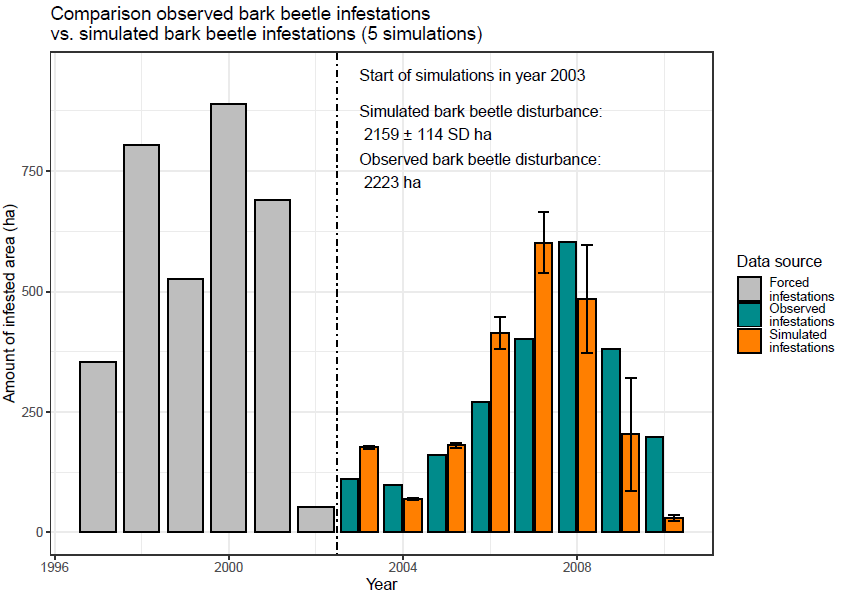


Supplemental Figure 1: Comparison of observed and simulated temporal pattern of bark beetle infestations. Simulated bark beetle infestations were forced to follow observed infestation until year 2002 in order to initialize bark beetle population levels. From 2003 onwards bark beetle dynamics was simulated as an emergent property in iLand. Simulated values are the annual mean area of bark beetle infestations derived from 5 replicated simulations, with error bars indicating their standard deviation.

| A) | B) |
| --- | --- |
| 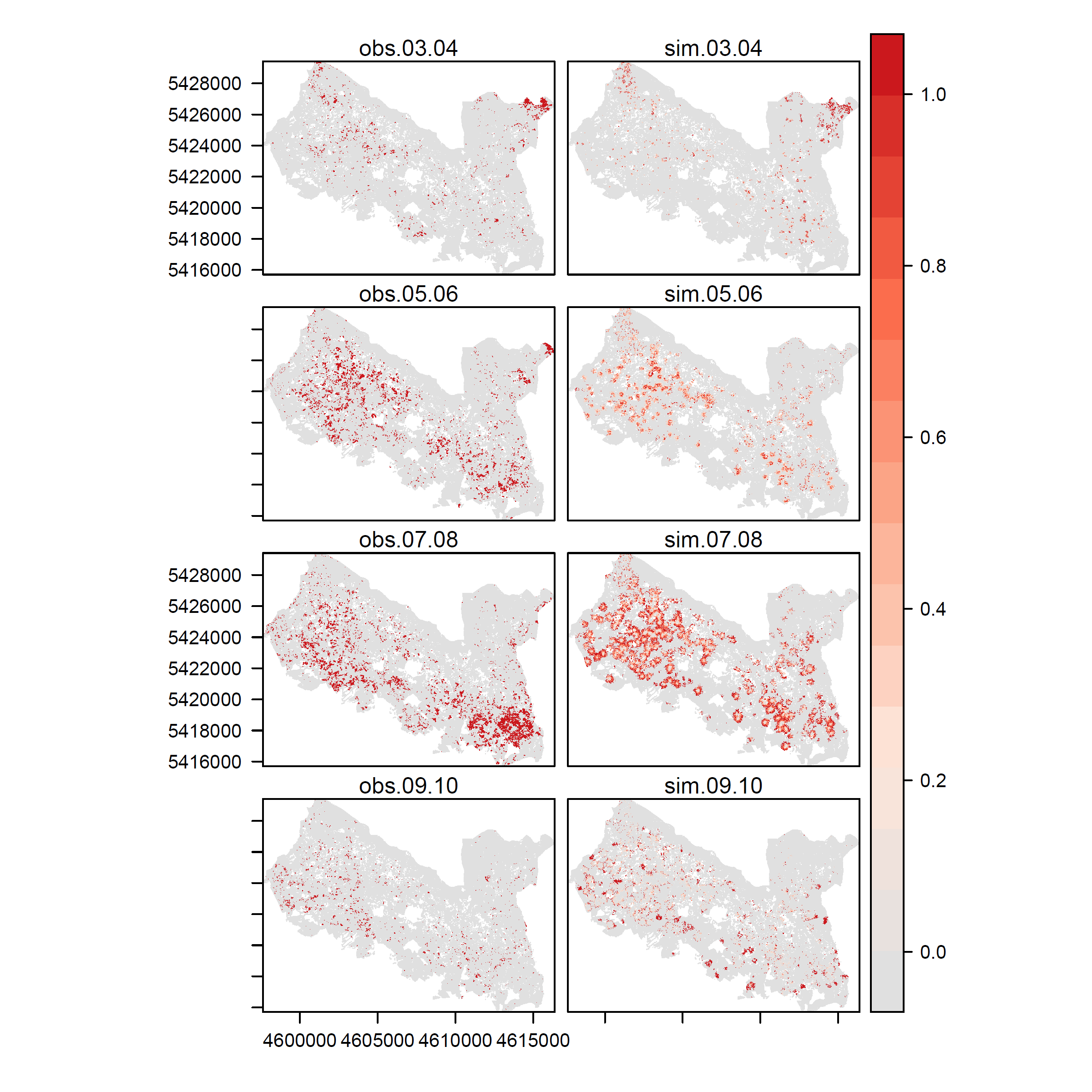 | 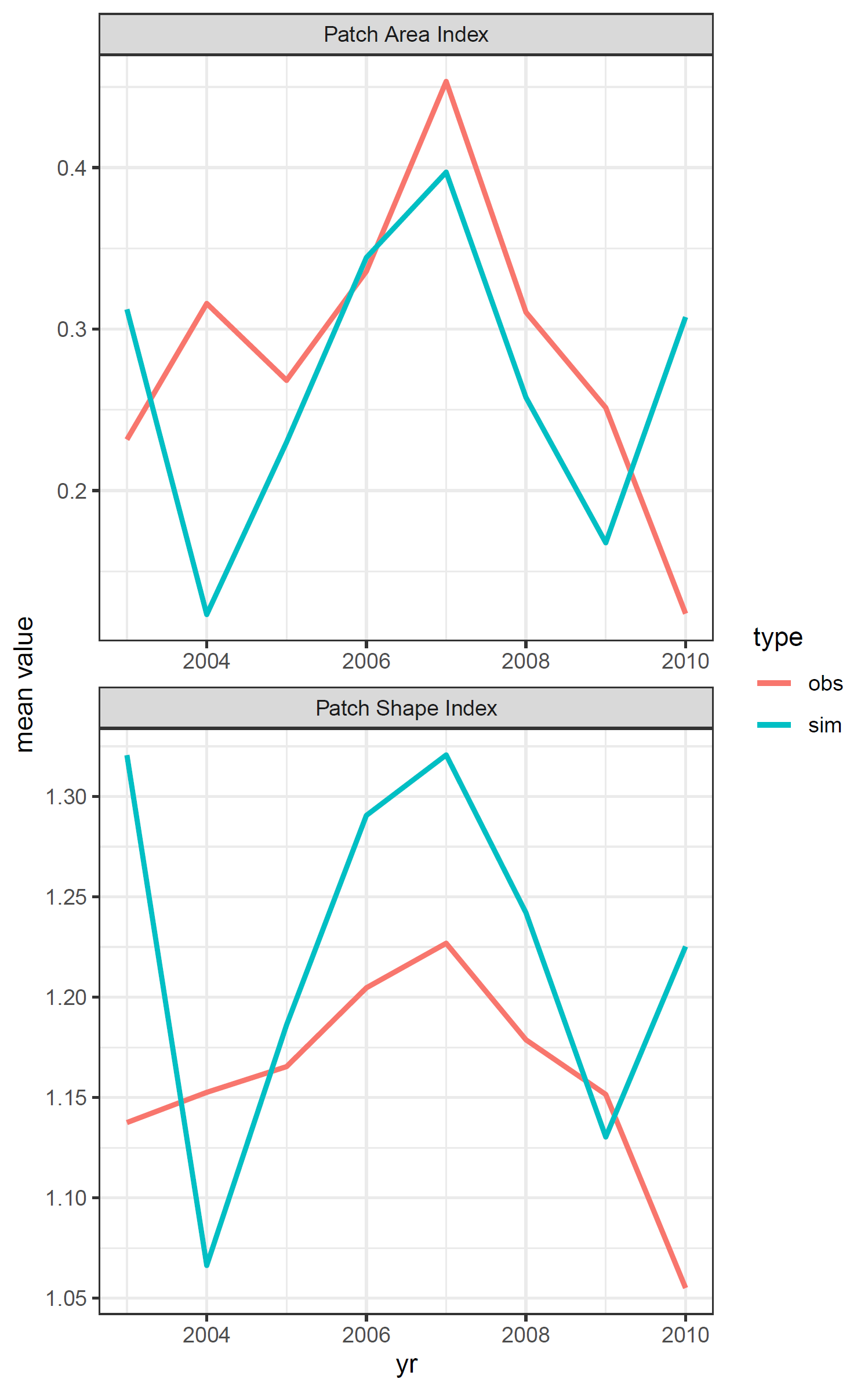 |

Supplementary Figure 2: A) Observed spatial patterns of bark beetle infestation (left) and simulated infestation probability based on five replicated simulations (right), aggregated in bins of two years for the period 2003 to 2010. The mean contiguity index over all years (± sd) was 0.124 ±0.035 for the observed outbreak and 0.094 ±0.032 for the simulated outbreak. B) Landscape metrics of the observed (obs) and simulated (sim) bark beetle outbreak, showing the mean patch area (ha) and the mean shape index (i.e., the ratio of actual perimeter to the hypothetical minimum perimeter of a disturbance of the same size) per year.


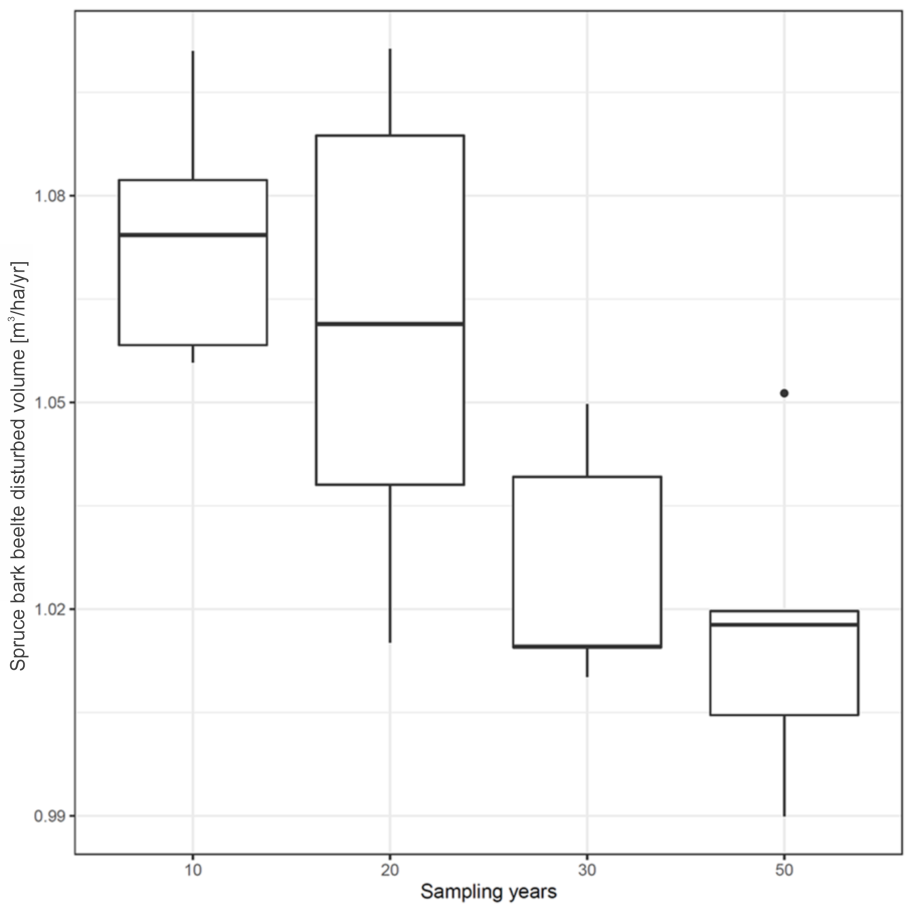


Supplementary Figure 3: Sensitivity of simulated bark beetle disturbances over the 600 year simulation period to different sampling periods used for extending the climate time series beyond the year 2100. The default value used in the main text is 30 years (i.e., sampling from the years 2070 – 2099 with replacement), and all values pertain to the HC scenario (RCP 8.5, ICHEC-EC-EARTH). Sampling from the last 10 and 20 years of the 21st century respectively increases simulated bark beetle disturbances by on average 3.9% and 2.9%, respectively. Sampling from the last 50 years of the century decreased simulated bark beetle disturbances by on average 1.0%. Boxes and whiskers give the variation from five replicated simulations with different years sampled.

# Productivity tests


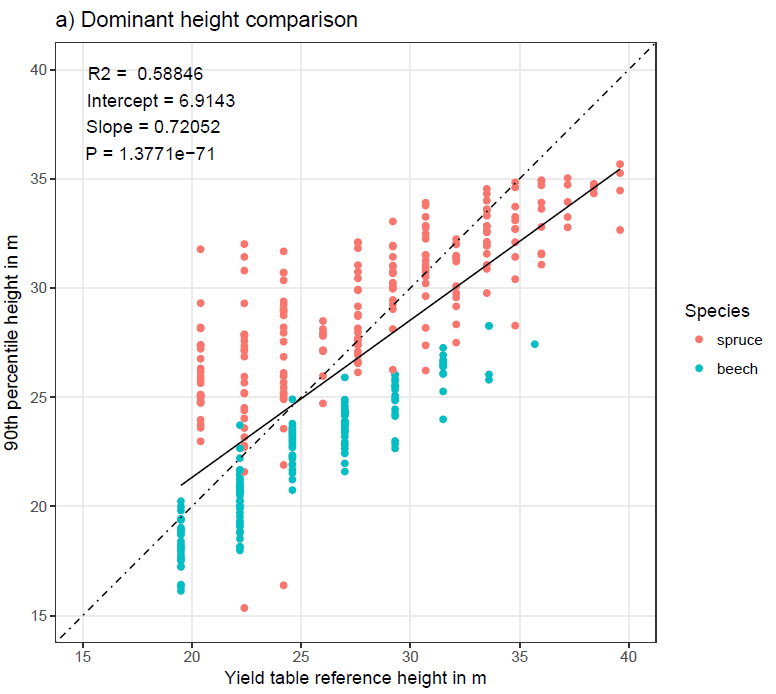


Supplemental Figure 4: Productivity comparison between the 90th percentile of predicted tree heights (y-axis) and reference dominant heights from suitable yield tables (x-axis). Tests were conducted in 401 single species stands at the age of 100 years. Samples were randomly selected from an elevation stratified subset of the two major tree species in Bavarian Forest National Park.


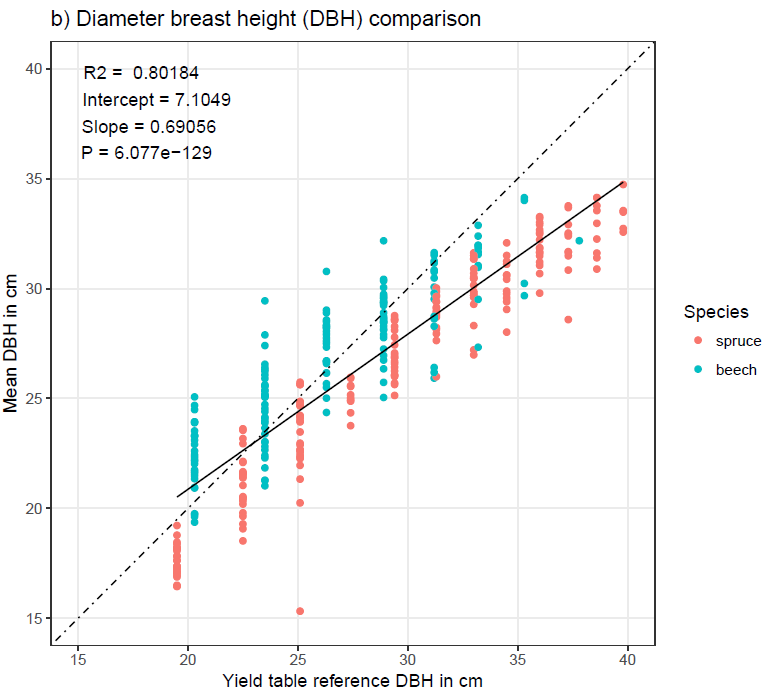


Supplemental Figure 5: Productivity comparison between predicted diameter at breast height (DBH) values (y-axis) and reference DBH values from suitable yield tables (x-axis). Tests were conducted in 401 single species stands at the age of 100 years. Samples were randomly selected from an elevation stratified subset of the two major tree species in Bavarian Forest National Park.

# Potential natural vegetation (PNV) tests

Supplemental Figure 6: Expected potential natural vegetation based on plant sociological assessments. Forests with 60% or more spruce or beech on total stand basal area were categorized as spruce or beech dominated forests.

Supplemental Figure 7: Simulated potential natural vegetation after 2000 simulated years. The simulation was started from bare ground and was run under baseline climate conditions and in the absence of disturbances. Forests with 60% or more spruce or beech on stand basal area were categorized as spruce or beech dominated forests.

Supplemental Figure 8: Comparison of forest area shares of different forest types between expected and simulated potential natural vegetation. Forests with 60% or more spruce or beech were categorized as spruce or beech dominated forests.


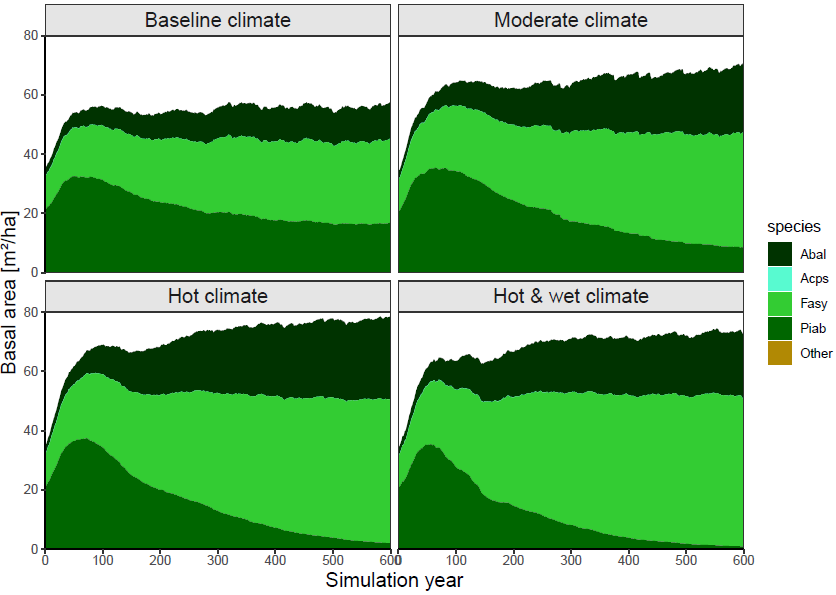
Supplemental Figure 9: Species shares under different scenarios of climate change (species: Abal = Abies alba, Acps = Acer pseudoplatanus, Fasy = Fagus sylvatica, Piab = Picea abies, Other = remaining species).

**Climate scenarios**

Supplemental Table 1: Description of simulated climate scenarios and their mean annual temperature and mean annual precipitation.

| Climate scenario name | Representative concentration pathway | Climate model | Mean annual temperature | Mean annual precipitation |
| --- | --- | --- | --- | --- |
| Base climate | Historic | Historic | 6.2 ° C | 1403 mm |
| Moderate climate change scenarios | 4.5 | ICHEC-EC-EARTH | 7.9 ° C | 1494 mm |
| Hot climate change scenario | 8.5 | ICHEC-EC-EARTH | 9.6 ° C | 1595 mm |
| Hot and wet climate change scenario | 8.5 | MOHC-HadGEM2-ES | 10.9 ° C | 1731 mm |

**Forest indicator changes for different climate scenarios**


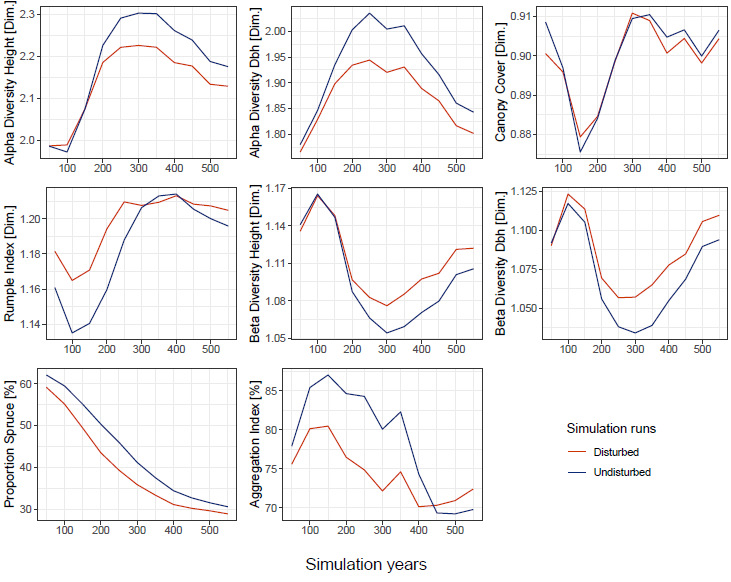


Supplemental Fig. 10: Effect of forest disturbance on forest structure and composition. Shown are the mean effects over all replicates under the baseline climate scenarios (BC). We refer to Table 1 for a detailed description of the indicators used.

Supplemental Table 2: Response of forest structure and composition to climate change and disturbances. Results are shown for the baseline climate scenarios (BC).

| Indicator | Attribute | Scale | Effect of climate change (*undisturbed simulations)* | Effect of disturbances (*disturbed simulations under BC climate*) | Effect of disturbances and climate change |
| --- | --- | --- | --- | --- | --- |
| α-diversity height | Structure | Stand | - | - 2.0 % | - |
| α-diversity dbh |  | Stand | - | - 2.8 % | - |
| Canopy cover |  | Landscape | - | - 0.2 % | - |
| Rumple index |  | Landscape | - | + 1.2 % | - |
| β-diversity height |  | Landscape | - | + 1.3 % | - |
| β-diversity dbh |  | Landscape | - | + 1.4 % | - |
| Proportion of spruce | Composition | Landscape | - | - 9.4 % | - |
| Aggregation index |  | Landscape | - | - 5.3 % | - |


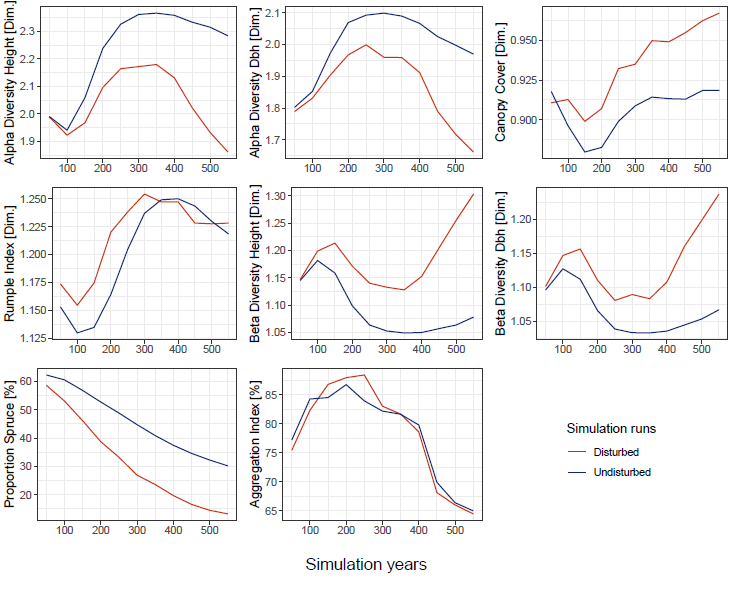


Supplemental Fig. 11: Effect of forest disturbance on forest structure and composition. Shown are the mean effects over all replicates under the moderate climate change scenarios (MC). We refer to Table 1 for a detailed description of the indicators used.

Supplemental Table 3: Response of forest structure and composition to climate change and disturbances. Results are shown for the moderate climate change scenario (MC).

| Indicator | Attribute | Scale | Effect of climate change (*undisturbed simulations)* | Effect of disturbances (*disturbed simulations under BC climate*) | Effect of disturbances and climate change |
| --- | --- | --- | --- | --- | --- |
| α-diversity height | Structure | Stand | + 2.3 % | - 2.0 % | - 6.6 % |
| α-diversity dbh |  | Stand | + 4.0 % | - 2.8 % | - 3.3 % |
| Canopy cover |  | Landscape | + 0.6 % | - 0.2 % | + 3.8 % |
| Rumple index |  | Landscape | + 1.5 % | + 1.2 % | + 2.9 % |
| β-diversity height |  | Landscape | - 0.7 % | + 1.3 % | + 8.0 % |
| β-diversity dbh |  | Landscape | - 0.7 % | + 1.4 % | + 5.8 % |
| Proportion of spruce | Composition | Landscape | + 4.1 % | - 9.4 % | - 28.5 % |
| Aggregation index |  | Landscape | - 0.3 % | - 5.3 % | - 0.1 % |


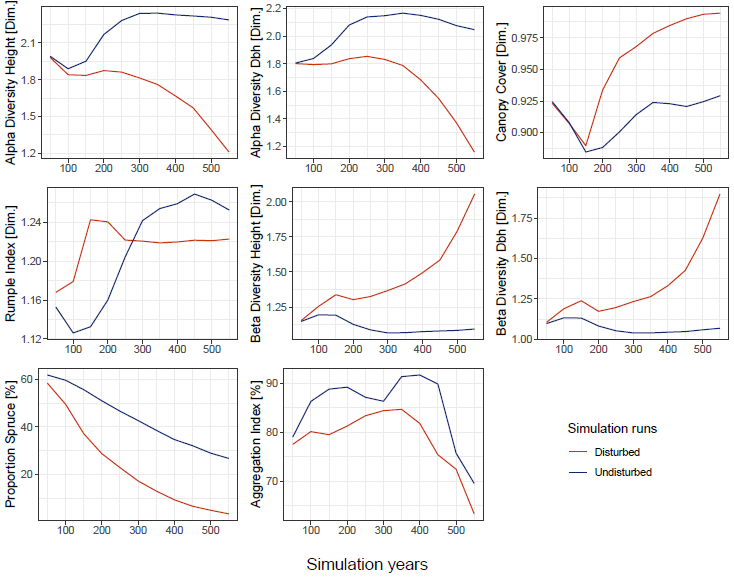


Supplemental Fig. 12: Effect of forest disturbance on forest structure and composition. Shown are the mean effects over all replicates for the hot climate change scenario (HC). We refer to Table 1 for a detailed description of the indicators used.

Supplemental Table 4: Response of forest structure and composition to climate change and disturbances. Results are shown for the hot climate change scenarios (HC).

| Indicator | Attribute | Scale | Effect of climate change (*undisturbed simulations)* | Effect of disturbances (*disturbed simulations under BC climate*) | Effect of disturbances and climate change |
| --- | --- | --- | --- | --- | --- |
| α-diversity height | Structure | Stand | + 0.8 % | - 2.0 % | - 21.7 % |
| α-diversity dbh |  | Stand | + 6.2 % | - 2.8 % | - 12.9 % |
| Canopy cover |  | Landscape | + 1.4 % | - 0.2 % | + 6.3 % |
| Rumple index |  | Landscape | + 2.3 % | + 1.2 % | + 2.8 % |
| β-diversity height |  | Landscape | + 1.1 % | + 1.3 % | + 33.2 % |
| β-diversity dbh |  | Landscape | + 0.0 % | + 1.4 % | + 24.5 % |
| Proportion of spruce | Composition | Landscape | - 0.6 % | - 9.4 % | - 47.8 % |
| Aggregation index |  | Landscape | + 8.2 % | - 5.3 % | ± 0.0 % |
